# Circ_0084615 promotes epithelial‐mesenchymal transition‐mediated tumor progression in hepatocellular carcinoma

**DOI:** 10.1002/ags3.12828

**Published:** 2024-06-03

**Authors:** Yu Wu, Li Peng

**Affiliations:** ^1^ Department of Surgical Teaching and Research Hebei Medical University Shi Jiazhuang Hebei China; ^2^ Department of Hepatobiliary Surgery The Fourth Hospital of Hebei Medical University Shi Jiazhuang Hebei China

**Keywords:** actin‐like 6A, circRNA, epithelial‐mesenchymal transition, hepatocellular carcinoma, migration

## Abstract

**Aim:**

CircRNAs have been identified as crucial regulators in tumorigenesis and progression. This study aimed to explore the biological role and underlying mechanism of circ_0084615 in hepatocellular carcinoma (HCC).

**Methods:**

The expression of RNAs was detected by quantitative reverse transcription‐polymerase chain reaction (qRT‐PCR). The effects of circ_0084615 silencing on malignant behaviors of HCC cells were assessed by CCK‐8, colony formation, wound healing, and Transwell assays in vitro and tumor transplantation experiment in vivo. The expression of proteins was detected by Western blotting. Dual‐luciferase reporter assay and RNA‐binding protein immunoprecipitation were performed to explore the mechanism of circ_0084615.

**Results:**

A significant upregulation of circ_0084615 was observed in HCC tissues, and positively correlated with the TNM staging. Silencing of circ_0084615 impeded HCC cell viability, colony formation, migration, invasion, epithelial‐mesenchymal transition, and xenograft tumor growth. Mechanistically, circ_0084615 could bind to miR‐1200 and eliminate its ability to destroy actin‐like 6A (ACTL6A) mRNA, thereby increasing ACTL6A expression and facilitating the malignant behaviors of HCC cells.

**Conclusions:**

This study clarified the oncogenic activity and mechanism of circ_0084615, thereby providing potential diagnostic biomarker and therapeutic target for inhibiting HCC progression.

## Graphical abstract

Circ_0084615 has identified as an oncogenic circRNA in hepatocellular carcinoma. circ_0084615 could bind to miR‐1200 and eliminate its ability to destroy actin‐like 6A (ACTL6A) mRNA, thereby facilitating the malignant behaviors of hepatocellular carcinoma cells in vitro and in vivo.

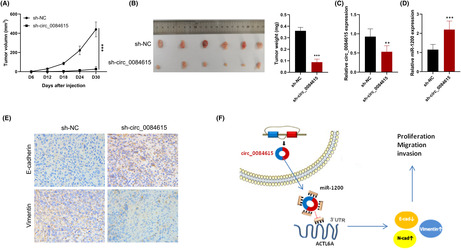

## 1 INTRODUCTION

Liver cancer is one of the most common malignant tumors and the third leading cause of cancer‐related death worldwide.^1^ Liver cancer includes hepatocellular carcinoma (HCC), intrahepatic cholangiocarcinoma and hepatocellular cholangiocarcinoma. HCC originates from hepatocytes, accounting for 70%–90% of all primary liver cancers. At present, the main clinical treatments for early HCC include local ablation, surgical resection, or liver transplantation. Only a small number of HCC patients are diagnosed at an early stage, and most are usually diagnosed at an advanced stage when the tumor is unresectable. However, there are limited therapeutic options available for HCC, and the clinical outcomes for some populations remain unsatisfactory.^2^ Therefore, it is necessary to further understand the pathogenesis of HCC and determine new diagnostic targets and therapeutic strategies.

CircRNAs are a class of non‐coding RNAs with a closed ring structure that exhibit more stability than linear RNAs.^3^ CircRNAs play regulatory roles in various physiological and pathological processes by acting as miRNA sponges, RNA‐binding protein (RBP) chaperones, or protein translation templates.^4^ The tissue‐specific and stage‐specific expression characteristics of circRNAs,^5^ making them ideal biomarkers for the diagnostic and prognostic prediction of many diseases, including HCC. For example, the combined panel including hsa_circ_00156, hsa_circ_000224, and hsa_circ_000520, showed superior performance characteristics relative to those of AFP, which could potentially be used in the diagnosis of HCC.^6^ Many circRNAs are involved in tumor formation, metastasis, recurrence, and chemical resistance.^7^, ^8^ Some cirRNAs, such as hsa_circ_0001445, hsa_circ_0001141, and circVAMP3, have been demonstrated to suppress the development of HCC.^9^, ^10^, ^11^ On the contrary, several cirRNAs, such as circRHOT1, circMDK, or circMRPS35, promote HCC tumorigenesis, progression or cisplatin resistance in HCC.^12^, ^13^, ^14^ These findings suggest the important role of circRNA in cancer progression and its potential for applications in cancer diagnosis and treatment. However, understanding the role and mechanism of circRNAs is still limited. It has been reported that circ_0084615 was upregulated in colorectal cancer (CRC), and high levels of circ_0084615 were closely associated with advanced tumor stage, lymph node metastasis, low differentiation, and poor prognosis in CRC patients. Further experiments demonstrated that circ_0084615 promoted CRC cell malignant behaviors by interacting with miR‐599 to elevate the levels of DNA methyltransferase 3 alpha (DNMT3A) or one cut homeobox 2 (ONECUT2).^15^, ^16^ A study reported that the expression of circ_0084615 was elevated in HCC samples by RNA sequencing.^17^ Importantly, our RT‐PCR test results further confirmed that hsa_circ_0084615 was indeed upregulated in HCC tissues compared with non‐cancer tissues in a cohort of 20 HCC patients, suggesting its possible involvement in HCC progression. Therefore, we further explored the possible role and molecular mechanism of circ_0084615 in HCC.

Here, functional experiments demonstrated that circ_0084615 silencing inhibited the malignant behaviors of HCC cells in vitro and tumor growth in vivo, implying the oncogenic activity of circ_0084615. Furthermore, we revealed that circ_0084615 could directly interact with miR‐1200, and eliminated its ability to disrupt the stability of the ACTL6A mRNA, thereby facilitating ACTL6A‐mediated malignant phenotypes of HCC cells. Together, these findings suggest the potential of circ_0084615 as a therapeutic target for HCC.

## 2 MATERIALS AND METHODS

### 2.1 Clinical specimens

Surgical specimens of patients with HCC were collected from the First Hospital of Qin Huangdao (Hebei, China) from January 2022 to December 2022. Fresh tissues were snap‐frozen in liquid nitrogen for subsequent RNA extraction.

### 2.2 Cell culture and transfection

BEL‐7405, MHCC97H, SK‐HEP‐2, Hep3B, and THLE‐2 cells were provided from Procell (Wuhan, China). Cells were cultured in DMEM, RPMI‐1640, or F–12 K mediums (Gibco, USA) according to instructions.

circ_0084615 shRNAs, miR‐1200 mimics, inhibitors, and the corresponding negative controls were provided by GenePharma (Suzhou, China). These plasmids were transfected into HCC cells with Lipofectamine 3000 (ThermoFisher, USA).

### 2.3 RNase R and quantitative real‐time PCR (QRT‐PCR)

Total RNA was extracted using the TRIzol reagent (Thermo, USA), and then reverse‐transcribed into cDNA using Advantage RT‐for‐PCR Kit (TaKaRa, Beijing, China). The qRT‐PCR analysis was performed using TB Green® Fast qPCR Mix (TaKaRa, Beijing, China). For RNase R treatment, total RNA was treated with RNase R (Merck, Germany) for 15 min at 37°C, 70°C for 5 min, and then was used for qRT‐PCR analysis. The primers circ_0084615 and miR‐1200 were provided by Sangon Biotech (Shanghai, China), and shown in Table 1.

**TABLE 1:** Primer sequence used in this study.

Gene	Sequence
hsa_circ_0084615	F: 5’‐AACTTATCAGAGGTGCTTCA‐3′ R: 5’‐CTACAGATGTCCAGACGC‐3′
linear_ 0084615	F: 5’‐GCAAAGGACTTCCGTTAT‐3′ R: 5’‐TTGGCATCATCCACATC‐3′
miR‐1200	RT: 5’‐GTCGTATCCAGTGCAGGGTCCGAGGTGCACTGGATACGACCTCCTGAG‐3’ F: 5’‐TGCGG GAGGCUCAGAAUGGCUCAGG‐3′
miR‐191	RT: 5’‐GTCGTATCCAGTGCAGGGTCCGAGGTGCACTGGATACGACCAACGGA‐3’ F: 5’‐TGCGG CAGCTGCTTTTGGGATTCCG‐3’
ACTL6A	F: 5’‐CAGAGCGGCTAAAGATTC‐3′ R: 5’‐CCTCCTGCCACTATTACACT‐3’

### 2.4 Fluorescence in situ hybridization (FISH)

To investigate the intracellular distribution of circ_0084615 in HCC cells, FAM‐labeled circ_0084615 probes were provided by GenePharma (Suzhou, China). When the cells grew to 70% confluence, cells were fixed with 4% paraformaldehyde. Fluorescence In Situ Hybridization Kit (GenePharma, China) was used for hybridization experiments according to the manufacturer's instructions. Images were obtained using a confocal microscope (Nikon, Japan).

### 2.5 CCK‐8 assay

CCK‐8 Kit (YEASEN, China) was used for the assessment of cell viability. BEL‐7405 and Hep3B cells (2 × 10^3^ cells per well) were cultured in a 96‐well plate. An amount of 10 μL CCK‐8 solution was added to each well. The 96‐well plates were placed at 37°C for 2 h. The absorbance at 450 nm was detected using a microplate reader.

### 2.6 Transwell assay

For the invasion assay, 5 × 10^4^ BEL‐7405 and Hep3B cells in serum‐free medium were added to the upper chamber (8 μm pore size, Corning) pretreated with Matrigel matrix. The lower chambers were added with 600 μL of medium containing 10% FBS. After incubation at 37°C for 24 h, the cells on the lower surface were fixed in 4% paraformaldehyde, stained with 0.05% crystal violet, photographed, and counted.

### 2.7 Western blot analysis

Antibodies used in Western blot were as follows: anti‐ACTL6A (1:1000, Proteintech, USA), AGO2 (1:1000, AHCCm, USA), anti‐E‐Cadherin (1:5000, Proteintech, USA), anti‐N‐Cadherin (1:2000, Proteintech, USA), anti‐Vimentin (1:2000, Proteintech, USA), and anti‐GAPDH (1:1000, Abclone, China).

### 2.8 Dual‐luciferase assay

The association of miR‐1200 with circ_0084615 or ACTL6A was assessed by dual‐luciferase reporter assay. The luciferase reporter vectors circ_0084615‐WT, circ_0084615‐MUT, ACTL6A‐WT, and ACTL6A‐MUT were constructed by introducing corresponding sequences into pmirGLO vectors (Promega, Madison, USA). These vectors and miR‐1200 or miR‐NC were co‐transfected into BEL‐7405 and Hep3B cells, followed by the analysis of the luciferase activity.

### 2.9 Animal experiments

BALB/c nude mice (5–6 weeks old) were provided by Beijing Vital River Laboratory Animal Technology Co., Ltd. (Beijing, China). The mice were randomly assigned to two groups. 5 × 10^5^ Hep3B cells transfected with sh‐circ_0084615 or sh‐NC were injected into the mice. Tumor volume was monitored every 6 days and calculated (length × width^2^ × 0.5). The mice were euthanized after 30 days and xenograft tumors were harvested for weight and immunohistochemistry.

### 2.10 Statistical analysis

Statistical analysis was performed by GraphPad Prism 8.0. Quantitative data were presented as mean ± standard deviation (SD). The difference between the two groups was assessed by the Student's t‐test. The difference among three or more groups was evaluated by ANOVA. *p* < 0.05 was considered statistically significant.

## 3 RESULTS

### 3.1 Elevated expression of circ_0084615 in HCC

**FIGURE 1:**
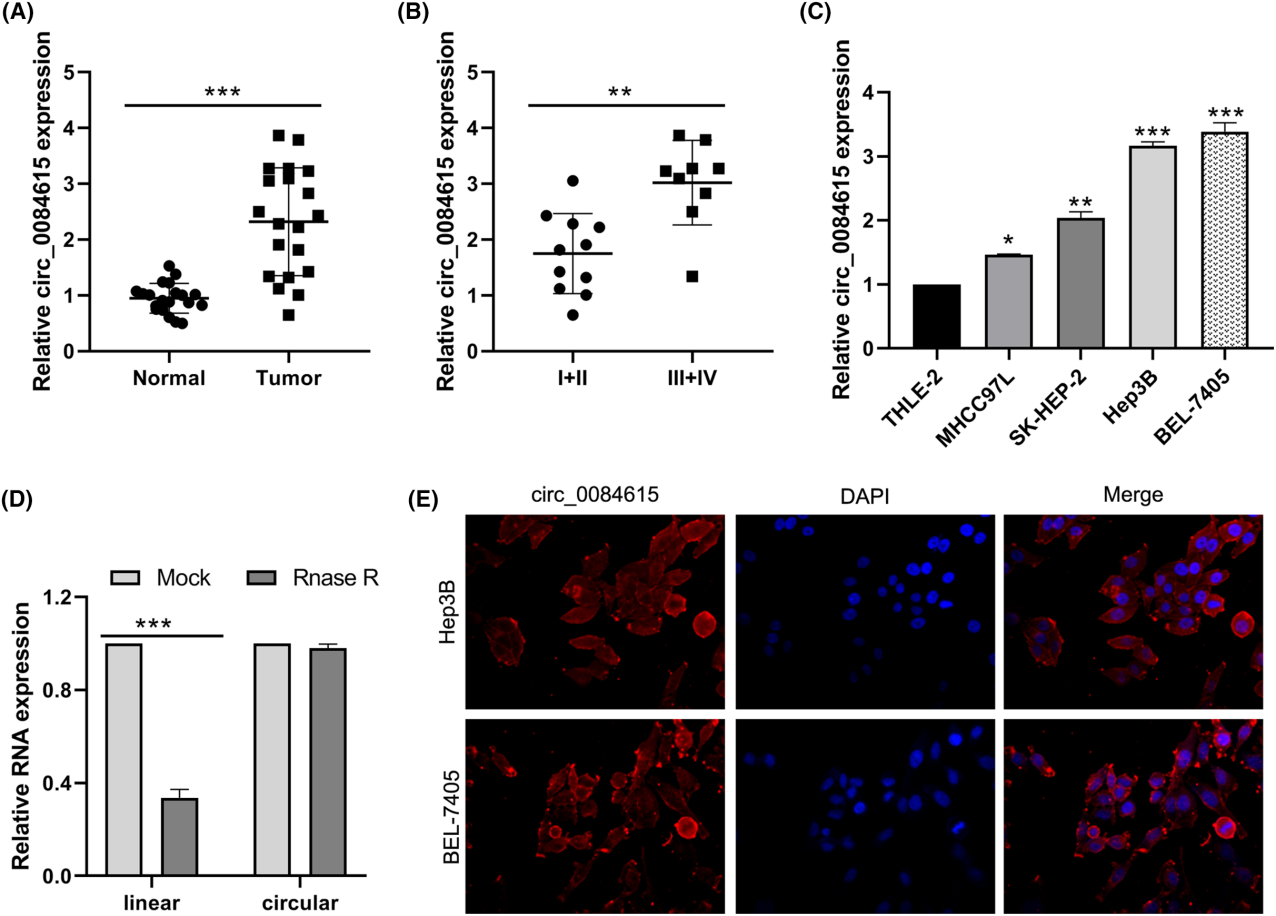
Elevated expression of circ_0084615 in HCC. (A) Expression of circ_0084615 in 20 pairs of HCC and adjacent normal specimens. (B) Expression of circ_0084615 expression in HCC tissues of I‐II and III‐IV tumor stages. (C) QRT‐PCR analysis of circ_0084615 in HCC cells and normal hepatocytes. (D) QRT‐PCR analysis of linear and circular transcripts in RNA treated with RNase R digestion. (E) FISH staining of circ_0084615 in BEL‐7405 and Hep3B cells. *p < 0.05, **p < 0.01, and ***p < 0.001.

QRT‐PCR results demonstrated that circ_0084615 was upregulated in HCC tissues compared to adjacent normal tissues (Figure 1A). The circ_0084615 was expressed at higher levels in high‐grade HCC than in low‐grade HCC (Figure 1B). Moreover, higher circ_0084615 levels exhibited an advanced tumor staging and portal vein tumor thrombus (Table 2). In addition, the expression of circ_0084615 was elevated in HCC cell lines (BEL‐7405, MHCC97H, SK‐HEP‐2, and Hep3B) relative to normal hepatocyte THLE‐2 cells (Figure 1C). Two HCC cell lines (BEL‐7405 and Hep3B) were selected for subsequent experiments due to the highest circ_0084615 levels among the four HCC cell lines.

**TABLE 2:** Relationships between circ_0084615 expression and clinicopathological characters of HCC patients.

Parameter	Numbers of patients	circ_0084615_high	circ_0084615_low	*p*‐value
Sex				
Female	7	1	6	0.057
Male	13	9	4	
TNM stage				
I + II	11	2	9	0.005
III + IV	9	8	1	
Tumor size (cm)				
<5	8	2	6	0.170
≥5	12	8	4	
Differentiation				
High+Middle	12	5	7	0.650
Low	8	5	3	
PVTT				
Negative	9	1	8	0.005
Positive	11	9	2	
Vessel invasion				
No	5	1	4	0.303
Yes	15	9	6	
Fibrosis				0.170
No	8	2	6	
Yes	12	8	4	

Abbreviation: PVTT, portal vein tumor thrombus.

In addition, the RNase R assay showed that RNase R treatment remarkably reduced the abundance of linear transcripts, but had no effect on circular transcripts (Figure 1D), suggesting that circ_0084615 was resistant to RNase R. Furthermore, the FISH experiment exhibited that circ_0084615 was primarily distributed in the cytoplasm of HCC cells (Figure 1E).

### 3.2 Knockdown of circ_0084615 inhibits the malignant behaviors of HCC

**FIGURE 2:**
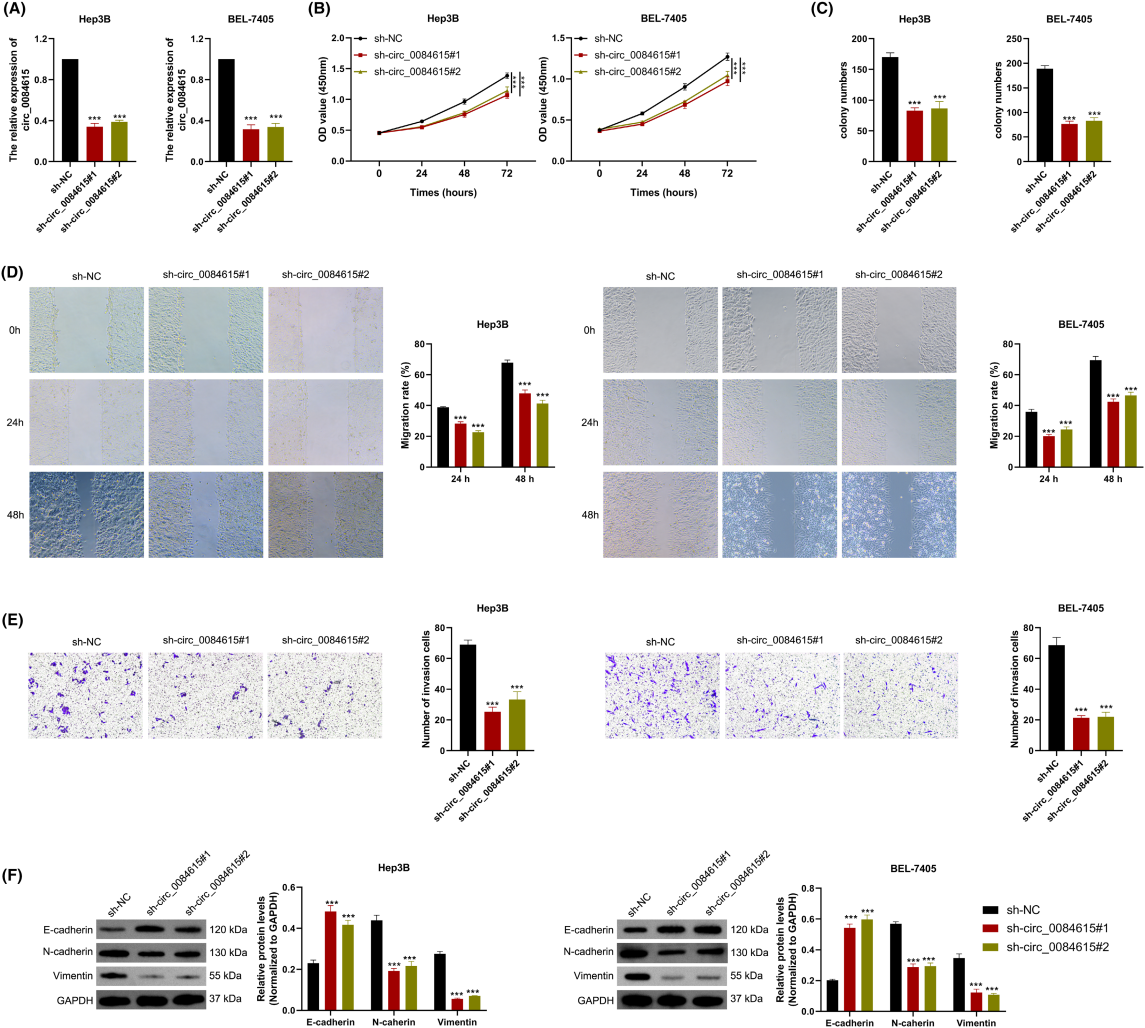
Inhibition of circ_0084615 on cell proliferation, migration, and invasion in HCC cells. (A) QRT‐PCR analysis of circ_0084615 levels in BEL‐7405 and Hep3B cells treated with circ_0084615 shRNA or negative control. (B) CCK‐8 assay was used for cell viability of HCC cells treated with circ_0084615 shRNA or negative control. (C) Colony formation assay was performed in HCC cells treated with circ_0084615 shRNA or negative control. (D) Wound healing assay was performed for cell migration. (E) Transwell assay was conducted for cell invasion of HCC cells treated with circ_0084615 shRNA or negative control. (F) Western blot analysis of the expression of E‐cadherin and N‐cadherin in BEL‐7405 and Hep3B cells transfected with circ_0084615 shRNA or negative control. ***p < 0.001 vs. the sh‐NC group.

To explore the function of circ_0084615 in HCC, two shRNAs were transfected into BEL‐7405 and Hep3B cells. QRT‐PCR assay confirmed that the two shRNAs (si‐1# and si‐2#) accurately and efficiently reduced circ_0084615 expression (Figure 2A). Subsequently, CCK‐8 experiment proved that circ_0084615 knockdown remarkably inhibited the proliferation ability of BEL‐7405 and Hep3B cells (Figure 2B). Likewise, in the colony formation experiment, we observed diminished colonies in circ_0084615‐silencing HCC cells (Figure 2C). Further, wound healing assay revealed that silenced circ_0084615 restrained the migration ability of BEL‐7405 and Hep3B cells (Figure 2D). Furthermore, Transwell assay indicated that silencing of circ_0084615 reduced the number of migrated BEL‐7405 and Hep3B cells (Figure 2E). Additionally, Western blot assay showed that circ_0084615 inhibition upregulated the epithelial marker E‐cadherin and downregulated the expression of the mesenchymal markers N‐cadherin and Vimentin (Figure 2F).

In addition, circ_0084615 expression was overexpressed in MHCC97L and SK‐HEP‐1 cells by circ_0084615‐overexpressing plasmids transfection (Figure S1A). CCK‐8 and colony formation assays showed that proliferation was increased after the elevated circ_0084615 expression in HCC cells (Figures S1B,C). Consistently, wound healing and Matrigel transwell assays showed that overexpression of circ_0084615 promoted the migration and invasion of MHCC97L and SK‐HEP‐1 cells (Figures S1D,E). Besides, overexpression of circ_0084615 downregulated the epithelial marker E‐cadherin and upregulated the expression of the mesenchymal markers N‐cadherin and Vimentin in MHCC97L and SK‐HEP‐1 cells (Figure S1F). Collectively, these results support that circ_0084615 enhances the proliferation, migration, and invasion of HCC cells.

### 3.3 circ_0084615 acts as a molecular sponge for miR‐1200

**FIGURE 3:**
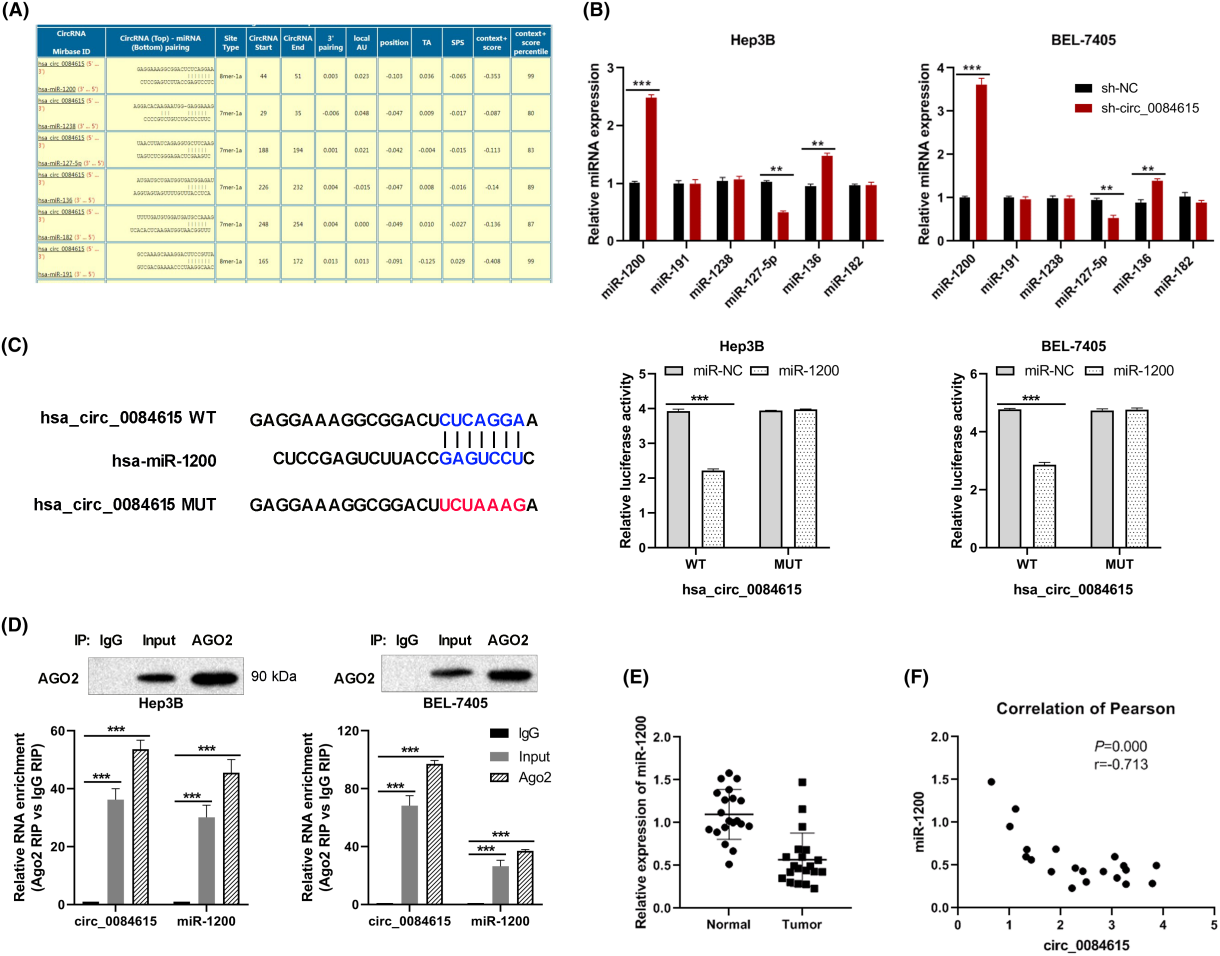
Circ_0084615 acts as a molecular sponge for miR‐1200. (A) Circular RNA Interactome was used to predict the potential miRNAs of has_circ_0084615. (B) QRT‐PCR analysis of miRNAs expression in BEL‐7405 and Hep3B cells treated with circ_0084615 shRNA or control. (C) The circ_0084615‐WT and MUT luciferase reporter vectors were constructed. Cells were co‐transfected with luciferase reporter vectors and the miR‐1200 mimic for 48 h, and the luciferase activity was detected. (D) RIP assay. (E) QRT‐PCR analysis of miR‐1200 expression in HCC and control tissues. (F) Pearson analysis for the correlation between circ_0084615 and miR‐1200. ***p < 0.001.

Considering that circ_0084615 was primarily distributed in the cytoplasm of HCC cells, we speculated that circ_0084615 may act as an miRNA molecular sponge in HCC cells. MiR‐1200, miR‐1238, miR‐127‐5p, miR‐136, miR‐182, and miR‐191 were predicted to interact with circ_0084615 by Circular RNA Interactome (Figure 3A). circ_0084615 deficiency significantly upregulated miR‐1200 in BEL‐7405 and Hep3B cells, and had slight or no effect on other miRNAs (Figure 3B). Luciferase reporter assay revealed that miR‐1200 mimics repressed the luciferase activity of circ_0084615‐WT plasmids but not mutant (Figure 3C). RIP experiment showed that both circ_0084615 and miR‐1200 could be enriched in the Ago2‐immunoprecipitation complex (Figure 3D). In addition, miR‐1200 levels in HCC tissues were lower than those from noncancerous samples (Figure 3E). Pearson analysis demonstrated that the expression of miR‐1200 was correlated negatively with circ_0084615 in HCC (Figure 3F). These results indicate that circ_0084615 directly binds to and inhibits miR‐1200 in HCC.

### 3.4 ACTL6A was a direct target of miR‐1200

**FIGURE 4:**
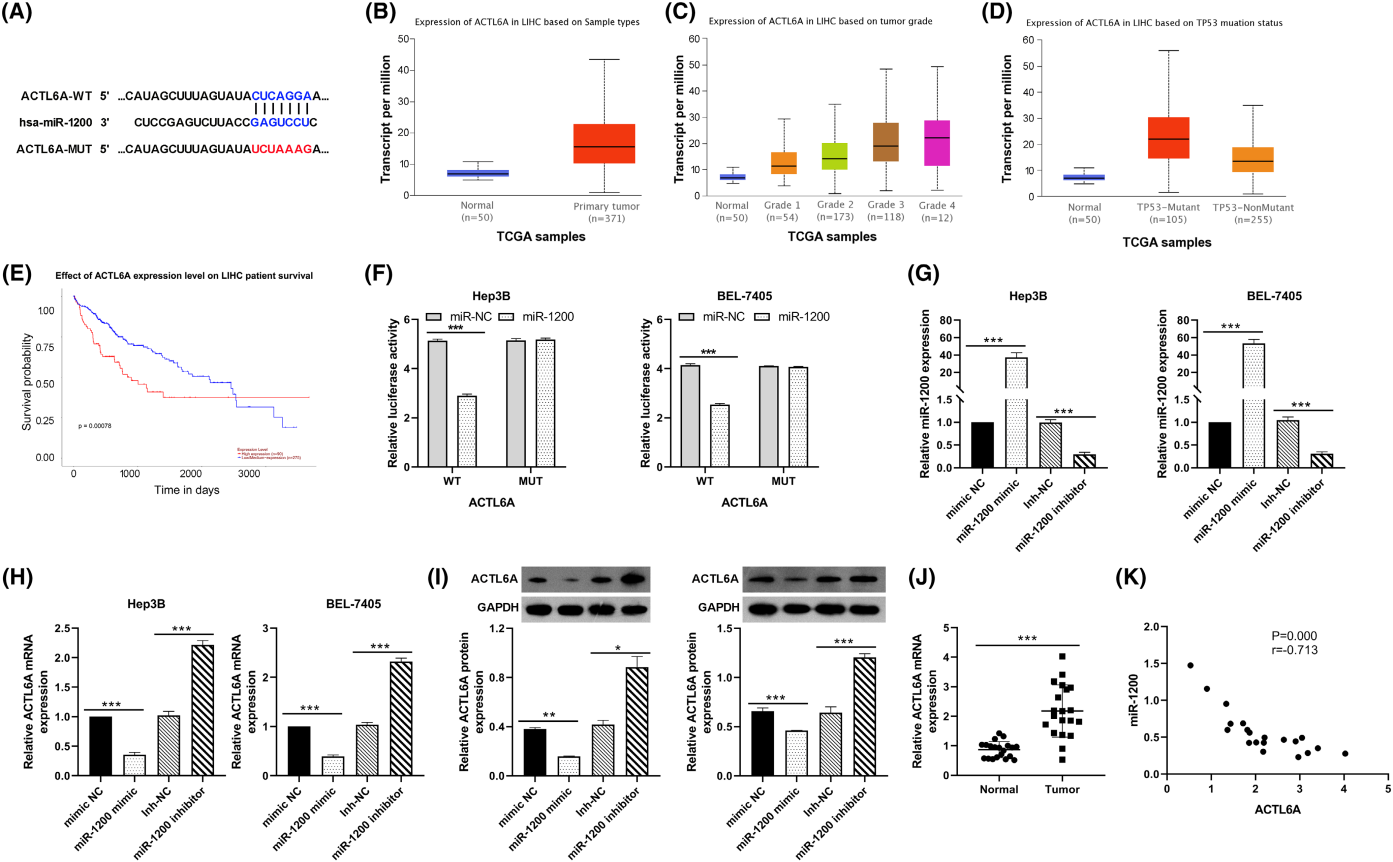
ACTL6A was a direct target of miR‐1200. (A) The predicted binding sequences between miR‐1200 and ACTL6A. (B) Analysis of ACTL6A expression in LIHC and normal specimens via the UALCAN database. (C) Analysis of ACTL6A expression in HCC tissues based on tumor grade via the UALCAN database. (D) Analysis of ACTL6A expression in HCC tissues based on tumor TP53 mutant status via UALCAN database. (E) Effect of ACTL6A expression level on HCC patients’ survival via UALCAN database. (F) Luciferase reporter assay to verify the binding of miR‐1200 to ACTL6A. (G) QRT‐PCR analysis of miR‐1200 expression in Hep3B and BEL‐7405 cells transfected with miR‐1200 mimic or inhibitor. (H) QRT‐PCR analysis of ACTL6A expression in Hep3B and BEL‐7405 cells transfected with miR‐1200 mimic or inhibitor. (I) Western blot analysis of ACTL6A expression in Hep3B and BEL‐7405 cells transfected with miR‐1200 mimic or inhibitor. (J) QRT‐PCR analysis of ACTL6A expression in HCC tissues. (K) Pearson analysis for the correlation between ACTL6A and miR‐1200. *p < 0.05, **p < 0.01, and ***p < 0.001.

TargetScan was used to predict the downstream mRNAs with potential binding sites for miR‐1200 (Figure 4A). It was found the ACTL6A harbored a potential miR‐1200 site. Previous studies demonstrated that ACTL6A controls the epithelial‐mesenchymal transition (EMT) progress in HCC cells and facilitates the invasive and migrative abilities of cancer cells.^18^ Through UALCAN database, it was found that the abundance of ACTL6A was remarkably increased in HCC tissues (*n* = 371) relative to the noncancerous samples (*n* = 50; Figure 4B). Notably, the abundance of ACTL6A was increased with the advanced tumor grade (Figure 4C). In particular, ACTL6A expression in TP53‐Mutant (*n* = 105) was higher than in TP53‐NonMutant tumors (*n* = 255; Figure 4D). Besides, high levels of ACTL6A predicts poor prognosis (Figure 4E). The luciferase reporter assay revealed that miR‐1200 reduced the luciferase activity of ACTL6A WT 3′ UTR, but not the mutant (Figure 4F). In BEL‐7405 and Hep3B cells, miR‐1200 mimic and inhibitor were used to overexpress and inhibit miR‐1200 expression, respectively (Figure 4G). Overexpression of miR‐1200 significantly downregulated ACTL6A expression, while miR‐1200 inhibitor showed opposite effects on ACTL6A protein (Figure 4H,I). Moreover, the expression of ACTL6A was upregulated in HCC tissues (Figure 4J) and correlated negatively with miR‐1200 in HCC tissues (Figure 4K). Overall, we identified ACTL6A as a potential regulatory target of circ_0084615/miR‐1200.

### 3.5 Inhibition of miR‐1200 diminished anti‐tumor activity mediated by circ_0084615 knockdown

**FIGURE 5:**
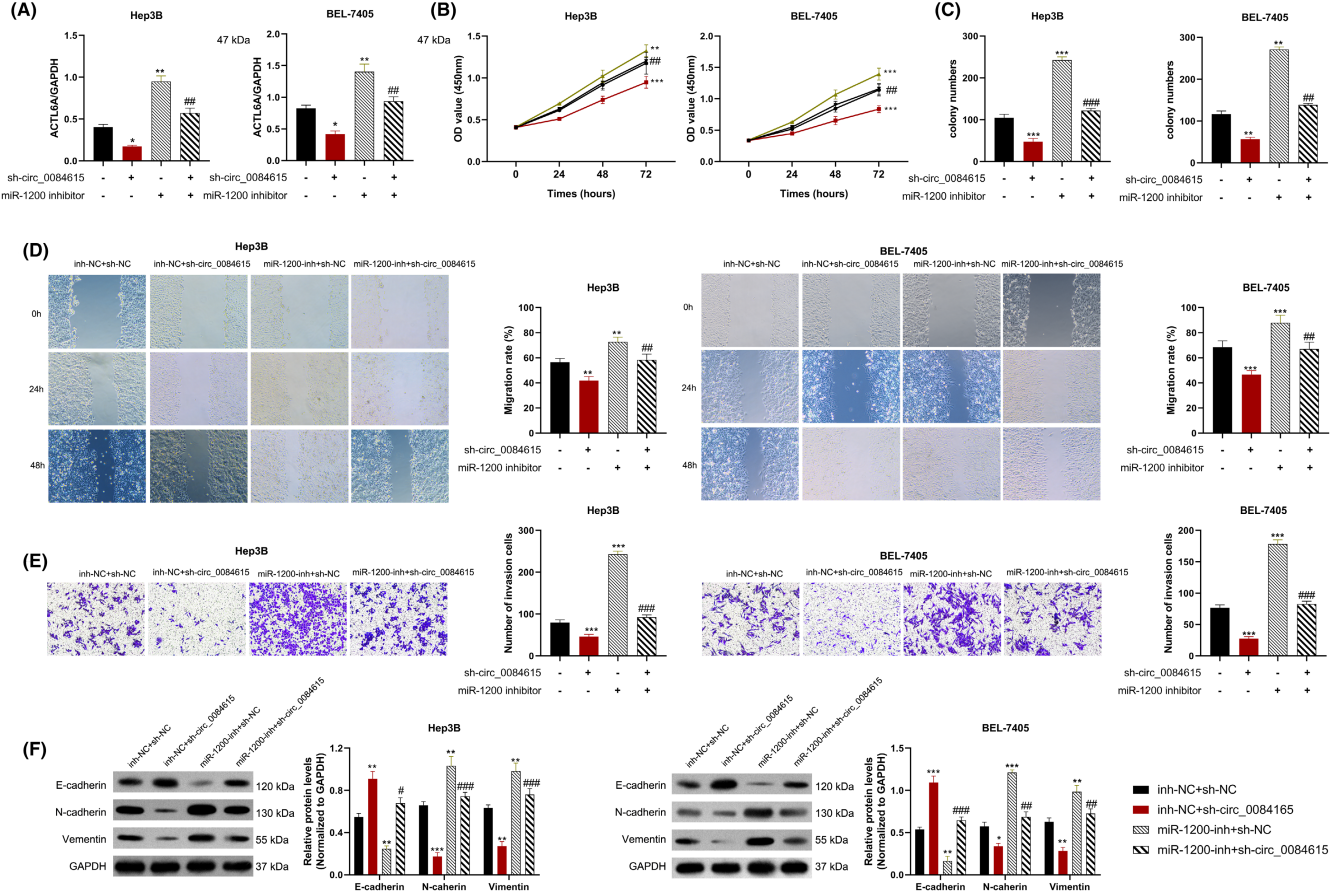
circ_0084615 promotes migration, and invasion of BEL‐7405 and Hep3B by downregulating miR‐1200. (A) Western blot analysis of ACTL6A expression with altered circ_0084615 and miR‐1200 expression. (B) CCK‐8 assay, (C) colony formation, (D) wound healing, and (E) transwell assays were performed in BEL‐7405 or Hep3B cells transfected with sh‐circ_0084615 and/or miR‐1200 inhibitor. (F) Western blot analysis of E‐cadherin, N‐cadherin, and Vimentin expression in BEL‐7405 and Hep3B cells transfected with sh‐circ_0084615 and/or miR‐1200 inhibitor. *p < 0.05, **p < 0.01, and ***p < 0.001 vs. the inh‐NC + sh‐NC group; ^#^ *p* < 0.05, ^##^ *p* < 0.01, and ^###^ *p* < 0.001 vs. the inh‐NC + sh‐circ_0084615 group.

We transfected BEL‐7405 and Hep3B cells with circ_0084615 shRNA plus miR‐1200 inhibitor. Compared with the negative control, circ_0084615 silencing led to the downregulation of ACTL6A expression, while miR‐1200 inhibitors upregulated its expression. However, co‐transfection reversed the effect of sh‐circ_0084615 silencing (Figure 5A). The decreased viability and colony formation by sh‐circ_0084615 were overturned and reversed by miR‐1200 inhibitors in Hep3B and BEL‐7405 cells (Figure 5B,C). Furthermore, wound healing and transwell assay experiments showed that circ_0084615 siRNA‐mediated decreased migration and invasion by sh‐circ_0084615 of both BEL‐7405 and Hep3B cells were attenuated by miR‐1200 inhibitors (Figure 5D,E). In addition, E‐cadherin upregulation and downregulation of N‐cadherin and Vimentin by circ_0084615 silencing were inverted in BEL‐7405 and Hep3B cells by miR‐1200 inhibitors (Figure 5F). In sum, we conclude that circ_0084615 enhances HCC cell migration and invasion through downregulating miR‐1200.

### 3.6 Downregulation of circ_0084615 reduces tumor growth and EMT in vivo

**FIGURE 6:**
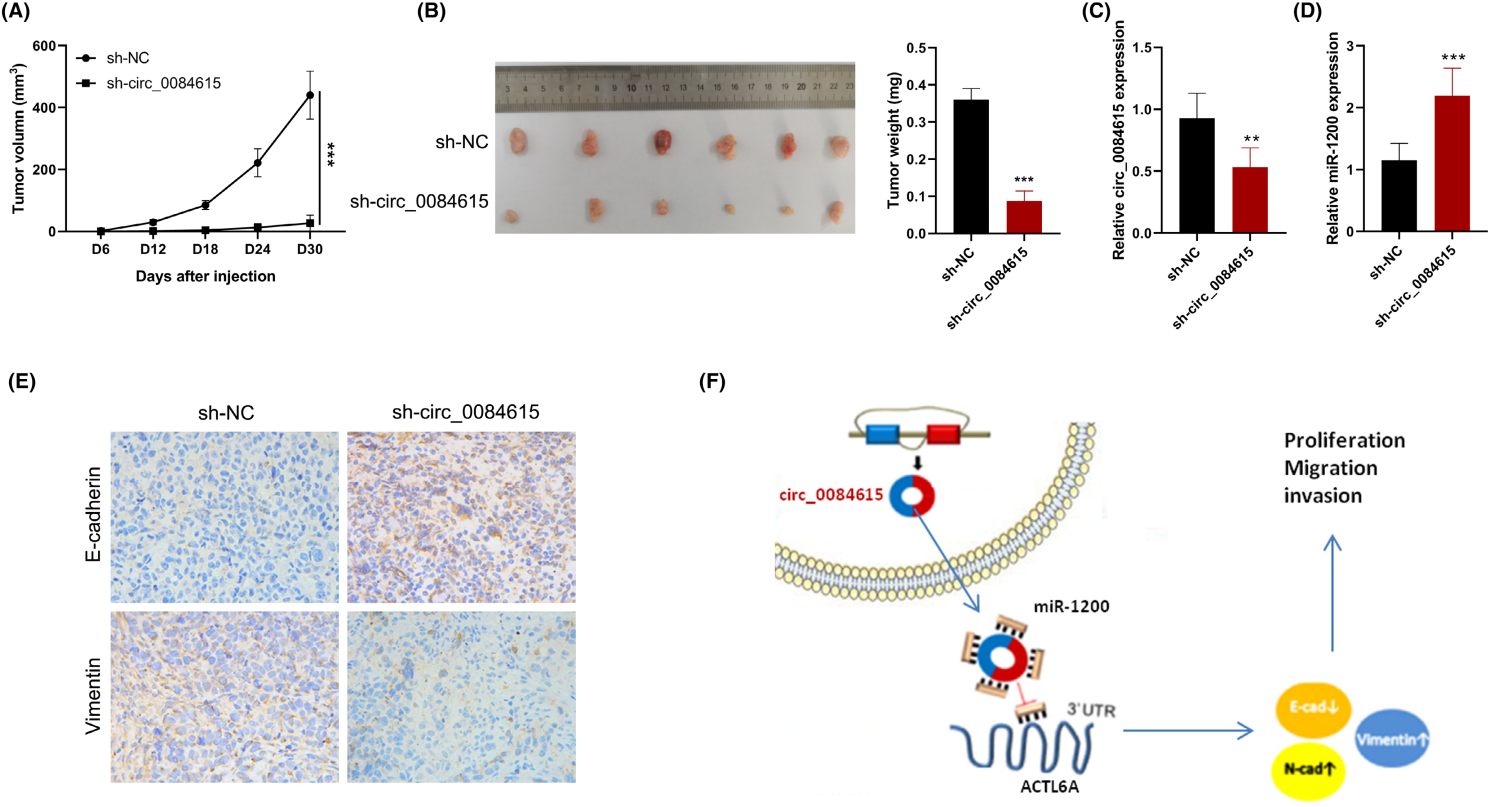
Downregulation of circ_0084615 reduces tumor growth and EMT in vivo. (A) Tumor volume. (B) Tumor weight. (C) The expression of circ_0084615. (D) The expression of miR‐1200. (E) Immunohistochemistry analysis of E‐cadherin and Vimentin. (F) Schematic representation of circ_0084615‐mediated oncogenic mechanisms in HCC. **p < 0.01, and ***p < 0.001 vs. the sh‐circ_0084615 group.

To evaluate whether targeted intervention with circ_0084615 affects tumor growth in vivo, we subcutaneously inoculated subjectively Hep3B cells with circ_0084615 shRNA plasmid ‐deficient, Hep3B cells with scrambled shRNA plasmid Hep3B cells into BALB/c nude mice. The results showed that tumor growth in the sh‐circ_0084615 group was lower than that in the negative control group (Figure 6A). Moreover, after 30 days, the xenografted tumor weight in sh‐circ_0084615 group was lighter than that of the negative control group (Figure 6B). Furthermore, the expression of circ_0084615 was reduced and miR‐1200 expression was increased in the sh‐circ_0084615 mice (Figure 6C,D). Additionally, the immunohistochemistry staining indicated that the expression of E‐cadherin was increased, and the expression of Vimentin was deceased by sh‐circ_0084615 (Figure 6E). These results suggest that downregulation of circ_0084615 significantly reduces tumor growth and EMT of HCC in vivo.

## 4 DISCUSSION

CircRNAs modulate the expression of many molecules by epigenetic, transcriptional, and posttranscriptional modification.^19^ A growing body of studies confirms the carcinogenic or tumor‐inhibiting effects of circRNA in HCC.^20^ The expression of circ‐AKT3, circ_0091579, and circ_0051040 was upregulated in HCC, and their silencing suppressed cell proliferation, migration, and invasion.^21^, ^22^, ^23^ Some circRNAs including circZKSCAN1 and circTMEM181 expression were down‐regulated in HCC, and their up‐regulation suppressed cell survival and metastasis.^24^, ^25^ In this research, we found that elevated circ_0084615 expression was associated with advanced TNM staging of HCC. Functional assays demonstrated that circ_0084615 promotes cell viability, migration, invasion, and EMT in vitro and in vivo. Besides, we revealed that circ_0084615 eliminates the ability of miR‐1200 to destroy ACTL6A mRNA, and thereby promoting tumor progression in HCC (Figure 6F). These findings indicate that circ_0084615 may be a promising therapeutic target for HCC treatment.

The molecular mechanism of circRNAs are related to their localization in the cells. In this study, circ_0085315 is mainly distributed in the cytoplasm. It has been reported that circRNAs mainly regulate mRNA stability or translation via absorbing miRNAs.^26^ Here, through the Circular RNA Interactome database, we explore potential miRNAs interacting with circ_0085315. Among the predicted targeted miRNAs, miR‐1200 has been reported to inhibit several oncogenes.^27^, ^28^, ^29^ In colon cancer, circ_0085315 adsorbs miR‐1200 to upregulate MAP3K1 and enhance cancer progression.^30^ CircZNF609 contributes to bladder cancer progression and decreases cisplatin sensitivity through the miR‐1200/CDC25B axis.^28^ Previous study showed that circ_0084615 could interact with miR‐599 to elevate the levels of ONECUT2 in CRC cells. However, the association between circ_0084615 and miR‐599 or ONECUT2 was not found in HCC cells (Figure S2). In our work, luciferase reporter assay confirmed the direct interaction between miR‐1200 and circ_0084615. Knockdown of circ_0084615 led to increased miR‐1200 expression in HCC cells. And, miR‐1200 was downregulated in HCC, and circ_0084615 was negatively correlated with miR‐1200 levels. Knockdown of circ_0084615 inhibited HCC cell migration, invasion, and EMT, which was reversed after miR‐1200 inhibition. These results indicate that circ_0084615 contributes to the malignant behaviors of HCC cells by regulation of miR‐1200.

To unveil the possible mechanism of miR‐1200 in HCC, we predicted the downstream mRNAs with potential binding sites for miR‐1200. ACTL6A was considered as a new target gene of miR‐1200. ACTL6A is a core subunit of the SWI/SNF chromatin remodeling complex. ACTL6A is highly expressed in several cancers and acts as a driver of cancer cell survival, differentiation, migration, and metastasis.^31^, ^32^, ^33^ Xiao et al.^18^ showed that upregulation of ACTL6A in HCC was associated with aggressive clinicopathological features, and predicted poor prognostic for HCC patients. ACTL6A enhances metastasis and EMT in HCC via the Notch pathway. Moreover, ACTL6A promoted HCC cancer stem cell‐like properties and tumorigenic abilities.^34^ We found that ACTL6A was significantly elevated in HCC tissues and negatively correlated with miR‐1200 expression. Overexpression of miR‐1200 negatively regulates the expression of ACTL6A, and their direct interaction was confirmed by Luciferase assay. In addition, the knockdown of circ_0084615 decreased ACTL6A expression, while the miR‐1200 inhibitor reversed this effect. These data support circ_0084615 enhanced HCC progression via the miR‐1200/ACTL6A axis.

## 5 CONCLUSION

The current study revealed that circ_0084615 accelerated HCC proliferation, migration, and invasion through the miR‐1200/ACTL6A axis. These findings elucidated the biological characteristics and mechanism of circ_0084615 and provided new therapeutic strategies in HCC.

## AUTHOR CONTRIBUTIONS

YW wrote the main manuscript, and prepared Figures 2, 3, 4, 5. LP collected clinical samples, conducted animal experiments, and prepared Figures 1 and 6. All authors read and approved the final manuscript.

## FUNDING INFORMATION

The authors received no financial support for the research, authorship, and/or publication of this article.

## CONFLICT OF INTEREST STATEMENT

The authors declared no potential conflicts of interest concerning the research, authorship, and/or publication of this article.

## ETHICS APPROVAL

Approval of the research protocols involving Animal and Human Studies in this present study was from the Ethics Committee of the First Hospital of Qinhuangdao (No. 2021Q077) and Informed Consent was obtained from the participants. This study was performed following the Helsinki Declaration of 1975, as revised in 2013.

## Supporting information


Figure S1.

Figure S2.

## Data Availability

The data used to support the findings of this article are available from the corresponding author upon request.

## References

[1] Sung H , Ferlay J , Siegel RL . Global cancer statistics 2020: GLOBOCAN estimates of incidence and mortality worldwide for 36 cancers in 185 countries. CA Cancer J Clin. 2021;71(3):209–249. 33538338 10.3322/caac.21660

[2] Gutiérrez‐Cuevas J , Lucano‐Landeros S , López‐Cifuentes D , Santos A . Epidemiologic, genetic, pathogenic, metabolic, epigenetic aspects involved in NASH‐HCC: current therapeutic. Strategies. 2022;15(1):23. 10.3390/cancers15010023 PMC9818030 36612019

[3] Chen T . Circulating non‐coding RNAs as potential diagnostic biomarkers in hepatocellular carcinoma. J Hepatocell Carcinoma. 2022;9:1029–1040. 36132427 10.2147/JHC.S380237 PMC9484560

[4] Wu Z , Yu X , Zhang S , He Y , Guo W . Mechanism underlying circRNA dysregulation in the TME of digestive system cancer. Front Immunol. 2022;13:951561. 36238299 10.3389/fimmu.2022.951561 PMC9550895

[5] Yepmo M , Potier JB , Pinget M , Grabarz A , Bouzakri K , Dumond Bourie A . Discussing the role of circular RNA in the pathogenesis of non‐alcoholic fatty liver disease and its complications. Front Endocrinol. 2022;13:1035159. 10.3389/fendo.2022.1035159 PMC9667057 36407314

[6] Matboli M , Shafei AE , Ali MA , Ashry AM , Kamal KM , Agag MA , et al. circRNAs (hsa_circ_00156, hsa_circ _000224, and hsa_circ _000520) are novel potential biomarkers in hepatocellular carcinoma. J Cell Biochem. 2019;120(5):7711–7724. 30426540 10.1002/jcb.28045

[7] Meng H , Niu R , Huang C , Li J . Circular RNA as a novel biomarker and therapeutic target for HCC. Int J Cancer. 2022;11(12):1948. 10.3390/cells11121948 PMC9222032 35741077

[8] Han Q , Wang M , Dong X , Wei F , Luo Y , Sun X . Non‐coding RNAs in hepatocellular carcinoma: insights into regulatory mechanisms, clinical significance, and therapeutic potential. Front Immunol. 2022;13:985815. 36300115 10.3389/fimmu.2022.985815 PMC9590653

[9] Yu J , Xu QG , Wang ZG , Yang Y , Zhang L , Ma JZ , et al. Circular RNA cSMARCA5 inhibits growth and metastasis in hepatocellular carcinoma. J Hepatol. 2018;68(6):1214–1227. 29378234 10.1016/j.jhep.2018.01.012

[10] Guo X , Wang Z , Deng X , Lu Y , Huang X , Lin J , et al. Circular RNA CircITCH (has‐circ‐0001141) suppresses hepatocellular carcinoma (HCC) progression by sponging miR‐184. Cell Cycle (Georgetown, Tex). 2022;21(15):1557–1577. 35400275 10.1080/15384101.2022.2057633 PMC9291649

[11] Chen S , Cao X , Zhang J , Wu W , Zhang B , Zhao F . circVAMP3 drives CAPRIN1 phase separation and inhibits hepatocellular carcinoma by suppressing c‐Myc Translation. Adv Sci. 2022;9(8):e2103817. 10.1002/advs.202103817 PMC8922094 35072355

[12] Du A , Li S , Zhou Y , Disoma C , Liao Y , Zhang Y , et al. M6A‐mediated upregulation of circMDK promotes tumorigenesis and acts as a nanotherapeutic target in hepatocellular carcinoma. Mol Cancer. 2022;21(1):109. 35524319 10.1186/s12943-022-01575-z PMC9074191

[13] Li P , Song R , Yin F , Liu M , Liu H , Ma S , et al. circMRPS35 promotes malignant progression and cisplatin resistance in hepatocellular carcinoma. Adv Sci. 2022;30(1):431–447. 10.1016/j.ymthe.2021.08.027 PMC8753434 34450251

[14] Wang L , Long H , Zheng Q , Bo X , Xiao X , Li B . Circular RNA circRHOT1 promotes hepatocellular carcinoma progression by initiation of NR2F6 expression. Mol Cancer. 2019;18(1):119. 31324186 10.1186/s12943-019-1046-7 PMC6639939

[15] Zhang B , Yang S , Wang J . Circ_0084615 is an oncogenic circular RNA in colorectal cancer and promotes DNMT3A expression via repressing miR‐599. Pathol Res Pract. 2021;224:153494. 34091391 10.1016/j.prp.2021.153494

[16] Jiang Z , Tai Q , Xie X , Hou Z , Liu W , Yu Z , et al. EIF4A3‐induced circ_0084615 contributes to the progression of colorectal cancer via miR‐599/ONECUT2 pathway. J Exp Clin Cancer Res. 2021;40(1):227. 34253241 10.1186/s13046-021-02029-y PMC8273970

[17] Liu Q , Cai Y , Xiong H , Deng Y , Dai X . CCRDB: a cancer circRNAs‐related database and its application in hepatocellular carcinoma‐related circRNAs. Database. 2019;2019:baz063. 31219565 10.1093/database/baz063 PMC6585150

[18] Xiao S , Chang RM , Yang MY , Lei X , Liu X , Gao WB , et al. Actin‐like 6A predicts poor prognosis of hepatocellular carcinoma and promotes metastasis and epithelial‐mesenchymal transition. Hepatology (Baltimore, Md). 2016;63(4):1256–1271. 10.1002/hep.28417 PMC4834727 26698646

[19] Shen H , Liu B , Xu J , Zhang B , Wang Y , Shi L , et al. Circular RNAs: characteristics, biogenesis, mechanisms and functions in liver cancer. J Hematol Oncol. 2021;14(1):134. 34461958 10.1186/s13045-021-01145-8 PMC8407006

[20] Louis C , Leclerc D , Coulouarn C . Emerging roles of circular RNAs in liver cancer. JHEP Rep. 2022;4(2):100413. 35036887 10.1016/j.jhepr.2021.100413 PMC8749337

[21] Dong B , Li H , Wang C , Qian X , Zhang R . Circ‐AKT3 promotes the proliferation and migration of HCC cells via down‐regulating microRNA‐335 expression. Minerva Med. 2022;113(6):1040–1041. 34477352 10.23736/S0026-4806.21.07715-6

[22] Ju L , Yao M . Circular RNA hsa_circ_0051040 promotes hepatocellular carcinoma progression by sponging miR‐569 and regulating ITGAV expression. Cells. 2022;11(22):3571. 36429000 10.3390/cells11223571 PMC9688127

[23] Mao Y , Ding Z , Jiang M , Yuan B , Zhang Y , Zhang X . Circ_0091579 exerts an oncogenic role in hepatocellular carcinoma via mediating miR‐136‐5p/TRIM27. Biom J. 2022;45(6):883–895. 10.1016/j.bj.2021.12.009 PMC9795369 34974169

[24] Lu B , Cao Li Q , Li Y Li Q , Lai H , Huang S , et al. circTMEM181 upregulates ARHGAP29 to inhibit hepatocellular carcinoma migration and invasion by sponging miR‐519a‐5p. Hepatol Res. 2023;53(4):334–343. 36519254 10.1111/hepr.13870

[25] Song R , Ma S , Xu J , Ren X , Guo P , Liu H , et al. A novel polypeptide encoded by the circular RNA ZKSCAN1 suppresses HCC via degradation of mTOR. Mol Cancer. 2023;22(1):16. 36691031 10.1186/s12943-023-01719-9 PMC9869513

[26] Liao W , Du J , Wang Z , Feng Q , Liao M , Liu H , et al. The role and mechanism of noncoding rnas in regulation of metabolic reprogramming in hepatocellular carcinoma. Int J Cancer. 2022;151(3):337–347. 35460073 10.1002/ijc.34040 PMC9325518

[27] Zhang Z . Circular RNA circ_0026359 enhances cisplatin resistance in gastric cancer via targeting miR‐1200/POLD4 pathway. Cell Biol Toxicol. 2020;2020:5103272. 10.1155/2020/5103272 PMC7443216 32855967

[28] Feng D , Lv J , Li K , Cao Q , Han J , Yu H , et al. CircZNF609 promotes bladder cancer progression and inhibits cisplatin sensitivity via miR‐1200/CDC25B pathway. Cell Biol Toxicol. 2022;39:1–18. 10.1007/s10565-022-09715-3 35567596

[29] Pan B , Zhao M , Wang N , Xu L , Wu T , Li Z . LncRNA RGMB‐AS1 promotes glioma growth and invasion through miR‐1200/HOXB2 Axis. Biomed Res Int. 2019;12:10107–10114. 10.2147/OTT.S230098 PMC6884071 31819505

[30] Luo Y , Yao Q . Circ_0085315 promotes cell proliferation, invasion, and migration in colon cancer through miR‐1200/MAP3K1 signaling pathway. Cell Cycle. 2022;21(11):1194–1211. 35230926 10.1080/15384101.2022.2044137 PMC9103513

[31] Zhang J , Zhang J , Wei Y , Li Q , Wang Q . ACTL6A regulates follicle‐stimulating hormone‐driven glycolysis in ovarian cancer cells via PGK1. Cell Death Dis. 2019;10(11):811. 31649264 10.1038/s41419-019-2050-y PMC6813335

[32] Shrestha S , Adhikary G , Xu W , Kandasamy S , Eckert RL . ACTL6A suppresses p21Cip1 expression to enhance the epidermal squamous cell carcinoma phenotype. Oncogene. 2020;39(36):5855–5866. 32616890 10.1038/s41388-020-1371-8 PMC7483332

[33] Jian Y , Huang X , Fang L , Wang M , Liu Q , Xu H , et al. Actin‐like protein 6A/MYC/CDK2 axis confers high proliferative activity in triple‐negative breast cancer. Oncogene. 2021;40(1):56. 10.1186/s13046-021-01856-3 PMC7863242 33541412

[34] Wang X , Li Y , Li Y , Liu P , Liu S , Pan Y . FBXW7 reduces the cancer stem cell‐like properties of hepatocellular carcinoma by regulating the ubiquitination and degradation of ACTL6A. Stem Cells Int. 2022;2022:3242482. 36159747 10.1155/2022/3242482 PMC9492413

