# Supplementary figures and images for: Circ_0084615 promotes epithelial‐mesenchymal transition‐mediated tumor progression in hepatocellular carcinoma
Source: Ann Gastroenterol Surg. 2024 Jun 3;8(6):1107–17. doi: 10.1002/ags3.12828 (PMC11533029; doi:10.1002/ags3.12828)

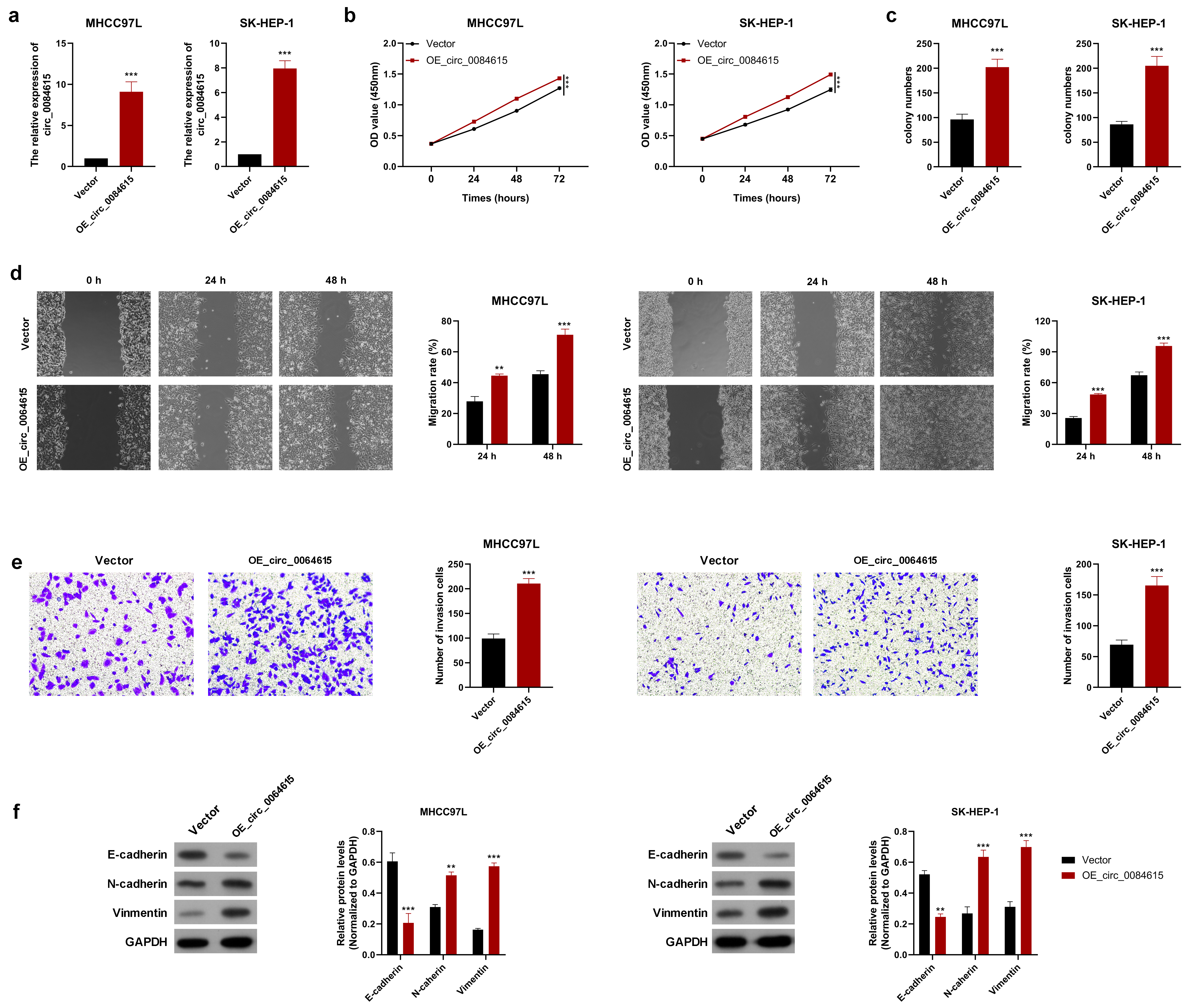

Supplement: Supplementary file 1 — Figure S1. [file AGS3-8-1107-s002.tif]

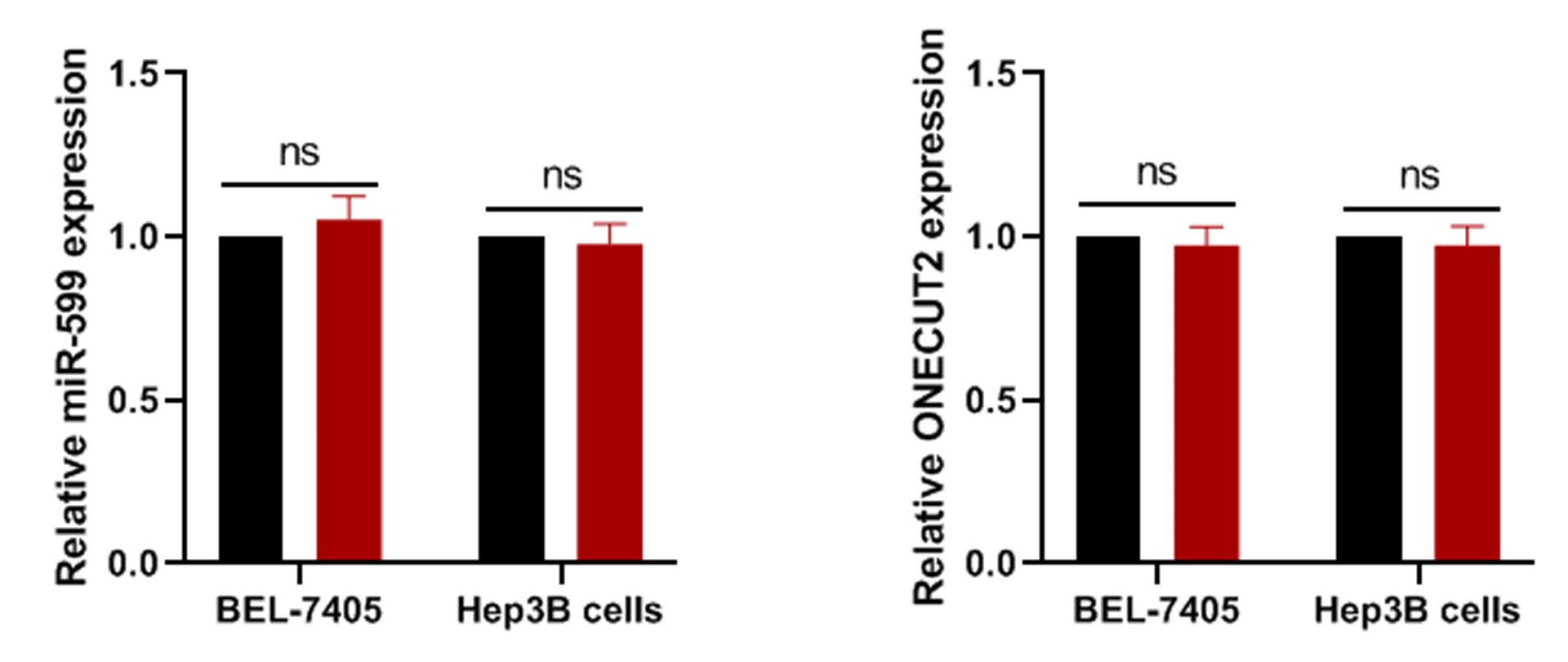

Supplement: Supplementary file 2 — Figure S2. [file AGS3-8-1107-s001.tif]
